# Long-Term Results of Multiple Pulmonary Metastasectomies

**DOI:** 10.1245/s10434-025-18992-1

**Published:** 2026-02-11

**Authors:** Michele Ferrari, Camilla Valsecchi, Giovanni Leuzzi, Luigi Rolli, Federica Sabia, Alessandro Pardolesi, Alessia Stanzi, Ugo Pastorino

**Affiliations:** 1 Division of Thoracic Surgery, Fondazione IRCCS Istituto Nazionale dei Tumori, Milan, Italy; 2 Lung Cancer Prevention Research, Fondazione IRCCS Istituto Nazionale dei Tumori, Milan, Italy

**Keywords:** Pulmonary metastasectomy, Lung metastases, Salvage surgery, Precision resection, Prognostic score, Risk factor

## Abstract

**Background:**

Pulmonary metastases affect up to 30% of cancer patients, but the incidence depends on the primary tumor type. Advances in therapy have expanded indications for pulmonary metastasectomy beyond highly selected cases. This study investigated long-term outcomes and prognostic factors of repeated pulmonary metastasectomies in modern clinical practice.

**Methods:**

This study analyzed the long-term results of 1503 pulmonary metastasectomies performed on 1106 consecutive patients at the Istituto Nazionale Tumori of Milan between 2003 and 2018, with a focus on multiple and repeated procedures, type of surgical resection, and key prognostic factors. Patients were stratified into three cohorts: single metastasis (SM, *n* = 530), multiple metastases (MM, *n* = 595), and recurrent metastases (RM, *n* = 378).

**Results:**

The 10 year survival rates were 35.5% overall, 41.1% for SM, 30.8% for MM, and 31.1% for RM. The median survivals were as 5.1, 5.4, 3.8, and 5.9 years respectively. Precision resection was the most common type of procedure (53.2%), with 54.0% survival at 5 years and 33.4% survival at 10 years. The 30 day postoperative mortality was 0.1% overall, 0% for SM, 0.3% for MM, and 0% for RM.

**Conclusions:**

These findings highlight the feasibility and curative potential of multiple and repeated pulmonary metastasectomies for carefully selected patients.

**Supplementary Information:**

The online version contains supplementary material available at 10.1245/s10434-025-18992-1.

About 30% of patients will experience pulmonary metastases, although the incidence depends on the primary tumor type,^1–6^ with an incidence of 9–34% for soft tissue sarcomas^7–10^ and 10–29% for colorectal cancer.^11,12^

During the last 20 years, the landscape of systemic therapy for metastatic tumors has changed significantly, expanding the number of available treatment options, and the choice of best therapy depends on tumor biology, response to target therapy, and metastatic burden.^13–15^

Historically, pulmonary metastasectomy (PM) was reserved for highly selected patients with limited disease burden, good performance status, and controlled primary tumors. However, over time, the indications for PM have expanded to include a broader cohort of patients. This shift has been driven by advancements in surgical techniques and development of more effective chemotherapeutic regimens, both of which have contributed to improve survival outcomes for metastatic patients.^16^

In 1991, the International Registry of Lung Metastases (IRLM) generated robust evidence that PM is not only a safe procedure but also potentially curative, with significant survival improvement. These findings have been supported by several observational studies, further reinforcing the role of PM in managing metastatic disease.^17^

Although the role of PM has been well-established in terms of favorable survival rates, the role of multiple and repeated PMs remains unclear, with only a few studies exploring this issue.^18,19^

More than 20 years after the IRLM results, this study aimed to further assess and potentially strengthen the evidence of efficacy in systematic PM. This report analyzes the overall survival at 10 years for patients undergoing multiple and repeated PMs to evaluate different resection techniques and validate the prognostic factors identified by the IRLM in the context of modern clinical practice.

## Methods

### Study Design and Population

We conducted a retrospective, observational, single-center analysis of patients who underwent a radical (R0) PM from January 2003 to December 2018 at the Fondazione IRCCS Istituto Nazionale dei Tumori (INT) of Milan. The analysis excluded patients with uncertain primary tumor site and diagnostic surgical procedures. The study enrolled 1106 patients.

The 1106 patients were categorized into three distinct cohorts based on their presentation at the time of the first procedure and the overall treatment strategy:

1. Single metastasis (SM) cohort: included patients who underwent a single resection with only one metastasis
2. Multiple metastases (MM) cohort: encompassed patients who presented with multiple metastases at the time of the first resection and subsequently underwent unilateral or sequential bilateral resections
3. Recurrent metastasectomies (MR) cohort: comprised patients who required multiple repeated metastasectomies for new recurrence episodes after a tumor-free period.

**Fig. 1:**
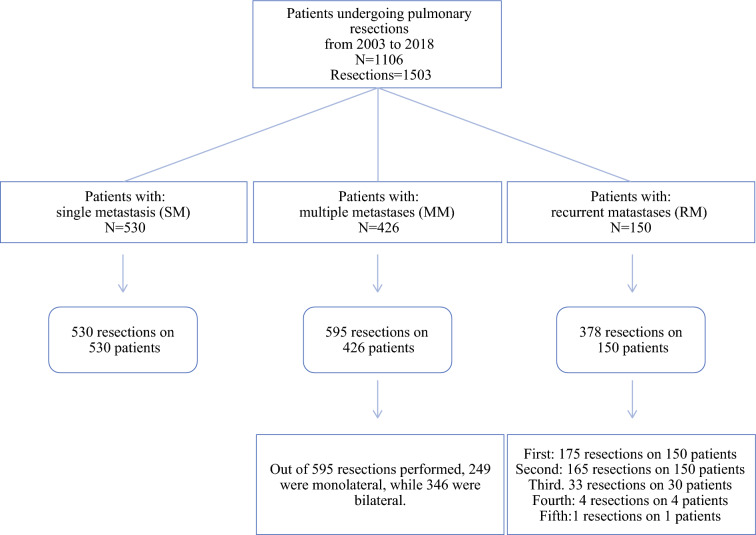
Flow-chart of the 1503 resections performed in 1106 patients

We classified patients based on the number of metastases at the first resection and the number of surgical procedures. Of the 1130 patients, 530 underwent single resection with only one metastasis (SM), 426 underwent monolateral or sequential bilateral resections with multiple metastases (MM) at presentation, and 150 underwent multiple repeated procedures for recurrence (RM), with single metastasis or multiple metastases at first resection (Fig. 1).

### Data Collection

The medical records were reviewed for demographic information, characteristics of primary tumor, and lung metastasectomy. Lung metastases were evaluated during staging of the primary tumor or during regular follow-up visits. In all cases, the preoperative workup included the execution of a whole-body computed tomography (CT) scan and a positron emission tomography (PET) scan to assess for recurrence or extra-thoracic disease to exclude patients who were not candidates for surgery. The PET scan was not performed for patients with nodules smaller than 6 mm or non– fluorodeoxyglucose (FDG)-avid tumors such as renal cell carcinoma or germ cell tumors.

All patients received preoperative systemic chemotherapy. The exceptions were patients with chemotherapy-refractory tumors (e.g., chondrosarcoma) and those with limited disease or a disease-free interval longer than 2 years after their prior adjuvant therapy. All lung nodules removed during the PM were histopathologically analyzed.

### Surgical Technique

The preferred technique adopted for metastasectomy was precision resection, performed by electrocautery or laser, to ensure complete tumor excision with adequate margins while preserving the surrounding parenchyma and limiting volumetric distortion compared with staplers. Parenchymal defects were closed by a single or bidirectional locked suture using 3-0 of polypropylene. In case of multiple resections, our policy was always to close the deeper defects, sometimes leaving the small superficial ones opened and covering them with aerostatic fibrin glues or patches. From March 2010 to December 2012, 66 consecutive patients were enrolled in a prospective clinical trial and received 80 laser precision resections and autologous fat tissue-grafting with the aim of preventing or reducing the air leaks.^20^

For selected patients with limited peripheral disease, metastasectomy was performed by mechanical staplers. In case of central lesions or patients with recurrent intra-lobar relapse, segmentectomy, lobectomy, or pneumonectomy was performed by open or thoracoscopic access. In the case of anatomic resections (segmentectomy, lobectomy, or pneumonectomy), systematic lymphadenectomy was performed, but lymphadenectomy was not routinely performed for non-anatomic resections such as precision or wedge resections. The choice between precision and wedge resection was based on a personalized strategy to balance oncologic radicality and lung parenchyma preservation. Wedge resection was preferred for solitary, peripheral lesions, whereas precision resection was our method of choice for multiple or centrally located lesions to maximize parenchymal preservation, which is critical for patients who may require repeated surgeries.

### Outcomes

The primary outcome to be assessed was overall survival (OS) at 5 and 10 years across the three study cohorts: SM, MM, and RM. The secondary outcomes were confirmation of prognostic factors such as disease-free interval (DFI) and total number of metastases as well as their impact on OS within the three cohorts, analysis of long-term OS by primary tumor site, validation of the IRLM prognostic system, evaluation of 30 and 90 day postoperative mortality across the three study cohorts, comparison of long-term OS stratified by resection type, assessment of the hospital length of stay stratified by the three cohorts, resection volume ,and surgical access. Disease-free interval was calculated from the date of surgical treatment of the primary tumor to the date of metastasis occurrences. Overall survival was calculated from the date of primary PM to death or last follow-up visit.

For the patients who underwent MM, OS was calculated from the date of first and second PM. The vital status was obtained through the Istituto Nazionale di Statistica (ISTAT; SIATEL 2.0 platform). The patients also were stratified into three prognostic groups according to the IRLM system based on the number of metastases and DFI: group 0 (no risk factor; DFI >36 months and single metastasis), group 2 (one risk factor; DFI <36 months or multiple metastases), group 3 (two risk factors; DFI <36 months and multiple metastases).^17^

### Statistical Analysis

Categorical data are presented as numbers and percentages, with the chi-square test used to find associations between the three classes. Continuous variables are presented as medians with interquartile ranges (IQRs), with the Wilcoxon-Mann–Whitney test used to test differences between classes. Overall survival at 5 and 10 years was estimated using the Kaplan-Meier method, and comparisons were tested by log-rank test. The median survival and the survival rates at 5 and 10 years were reported, stratified by all variables of interest. A multivariate Cox proportional hazard regression model was implemented to identify the risk factor for OS, stratified for the three cohorts. A generalized linear model (Poisson family and link-log) was performed to analyze which variables increase the probability of a longer hospital stay.

All *p* values lower than 0.05 were considered statistically significant. Analyses were performed using Statistical Analysis System Software (Release SAS: 9.04; SAS Institute, Cary, NC, USA)

## Results

The median follow-up time was 9.5 years for patients still alive at the end of the study (*n* = 396).

For the 1106 patients, 1503 resections were performed from January 2003 to December 2018. Of these 1106 patients, 530 (47.9%) underwent unilateral single resection with only one metastasis, 426 (38.5%) underwent a single resection (249 monolateral and 177 sequential bilateral) for multiple metastases, and 150 (13.5%) underwent 378 repeated PMs, with 30, 4, and 1 undergoing a third, fourth, and fifth resection, respectively (Fig. 1).

### Patients’ Characteristics

Among the 1106 patients, 41.4% (458/1106) were females and 58.6% (648/1106) males (*p* = 0.6088). Half of the patients (50.1%) were ages 50 to 69 years, whereas 19.3% (213/1106) were 70 years old or older. A higher proportion (64.3%) of patients older than 70 was observed in the SM cohort (*p* < 0.0001). The most common primary tumors were colon-rectum carcinoma (31.1%), sarcoma (28.8%), and melanoma (8.9%) (Table S1). Pulmonary resection was performed for 364 (32.9%) patients 36 months after the primary tumor. Among these patients, 33.5% had two IRLM risk factors, 46.3% had one IRLM risk factor, and only 20.2% had no IRLM risk factors (Table S1).

### Surgical Procedures

Of the 1503 resections performed, 1067 (71%) were monolateral and 436 (29%) were bilateral. The most common surgical access was limited muscle-sparing thoracotomy, accounting for 80.9% (1216/1503) of procedures, whereas video-assisted thoracic surgery (VATS) was applied to 18.1% (284/1503) of resections. We performed 799 (53.2%) precision resections, 284 (18.9) wedge resections, 223 (14.8%) lobectomies, 181 (12%) segmentectomies, and only 16 (1.1%) pneumonectomies.

All these variables differed statistically among the three cohorts (*p* < 0.0001). Half of the pneumonectomies (56.3%) were performed in the SM cohort, whereas half of the precision resections (52.9%) were performed in the MM cohort. In the RM cohort, 205 resections used a precision technique. A total of 823 resections removed only one metastasis, and 45 resections removed 10 or more metastases. In the MM group, 96 resections presented one metastasis, occurring in the sequential bilateral resection.

At the 90 day evaluation, we observed eight deaths (0.5%). In the SM group, three (0.6%) patients died within 90 days. Two of the three patients died after a wedge resection with a thoracotomy approach, and the remaining patient died after a precision resection with a VATS approach. In the MM group, four (0.7%) deaths occurred (1 after segmentectomy by sternotomy, 1 after precision resection by thoracotomy, 1 after wedge resection by thoracotomy, and 1 after pneumonectomy by thoracotomy). In the RM group, one (0.3%) death was observed after lobectomy by thoracotomy (Table 1).

**Table 1:** Characteristics of all resections performed, stratified by the three different study cohorts

	Total	SM	MM	RM	*p* Value
	(*n* = 1503) *n* (%)	(*n* = 530) *n* (%)	(*n* = 595) *n* (%)	(*n* = 378) *n* (%)	
Laterality					
Monolateral	1067 (71.0)	530 (49.7)	249 (23.3)	288 (27.0)	<0.0001
Bilateral	436 (29.0)	0	346 (79.4)	90 (20.6)	
First type of approach					
Thoracotomy	1216 (80.9)	363 (29.9)	519 (42.7)	334 (27.5)	<0.0001
VATS	272 (18.1)	164 (60.3)	68 (25.0)	40 (14.7)	
Other^a^	15 (1.0)	3 (20.0)	8 (53.3)	4 (26.7)	
Type of resection					
Precision resections	799 (53.2)	171 (21.4)	423 (52.9)	205 (25.7)	<0.0001
Wedge	284 (18.9)	165 (58.1)	46 (16.2)	73 (25.7)	
Lobectomy	223 (14.8)	109 (48.9)	59 (26.5)	55 (24.7)	
Segmentectomy	181 (12)	76 (42)	64 (35.4)	41 (22.7)	
Pneumonectomy	16 (1.1)	9 (56.3)	3 (18.8)	4 (25)	
Median hospital stay (days)					
<7	1162 (77.3)	421 (36.3)	459 (77.1)	282 (74.6)	0.2286
≥7	341 (22.7)	109 (31.8)	136 (22.9)	96 (25.4)	0.0032^b^
Median (IQR)	5 (4–6)	5 (3–6)	5 (4–6)	5 (4–7)	
No. of lesions removed					
1	641 (42.65)	468 (73.01)	na	173 (26.99)	<0.0001
2–4	609 (40.52)	58 (9.52)	403 (66.2)	148 (24.3)	
5–9	176 (11.71)	4 (2.3)	126 (71.6)	46 (26.1)	
10+	77 (5.1)	0	66 (85.7)	11 (14.3)	
No. of pathologic metastases^c^					
1	823 (54.8)	530 (64.4)	96 (11.7)	197 (23.9)	<0.0001
2–4	491 (32.7)	0	344 (70.1)	147 (29.9)	
5–9	137 (9.1)	0	113 (82.5)	24 (17.5)	
10+	45 (3)	0	38 (84.4)	7 (15.6)	
Pos operative mortality					
30 day	2 (0.1)	0	2 (0.3)	0	
90 day	8 (0.5)	3 (0.6)	4 (0.7)	1 (0.3)	

VATS, video-assisted thoracic surgery; IQR, interquartile range

^a^Other: clamshell, sternotomy, transmanubrial

^b^Wilcoxon-Mann–Whitney test

^c^We omitted 7 cases due to the complete pathologic remission after chemoradiotherapy

### Survival

The overall median survival was 5.1 years (95%confidence interval [CI], 4.6–5.4 years). The median survival, stratified by cohorts, was 5.4 years (95%CI, 5.0–5.8 years) for SM, 3.8 years (95%CI, 3.2–4.6 years) for MM, and 5.9 years (95%CI, 5.7–7.0 years) for RM. More than 10 years after the first PM, 215 patients were still alive. Of these patients, 34 (15.8%) underwent more than one PM.

**Fig. 2:**
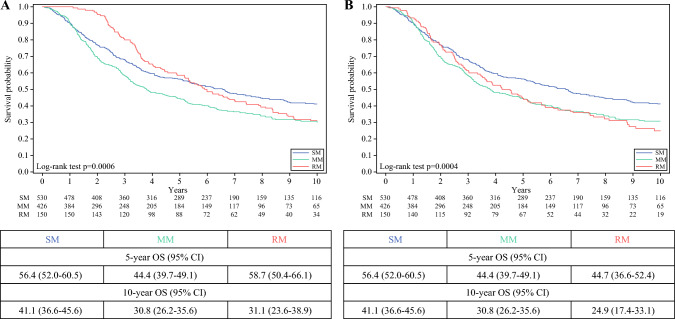
10 year overall survival according to the three cohorts (Panel A). For multiple resections group, follow-up started by the second resection (Panel B)

The 5 and 10 year OS rates after the first PM for the entire population (*n* = 1106) were 52.1%(95%CI, 49.1–55.0%) and 35.5%(95%CI, 32.5–38.5%), respectively. The 10 year OS rates for the three cohorts were as follows: 41.1%(95%CI, 36.6–45.6%) for SM, 30.8%(95%CI, 26.2–35.6%) for MM, and 31.1%(95%CI, 23.6–38.9%) for RM (Fig. 2, panel A).

**Fig. 3:**
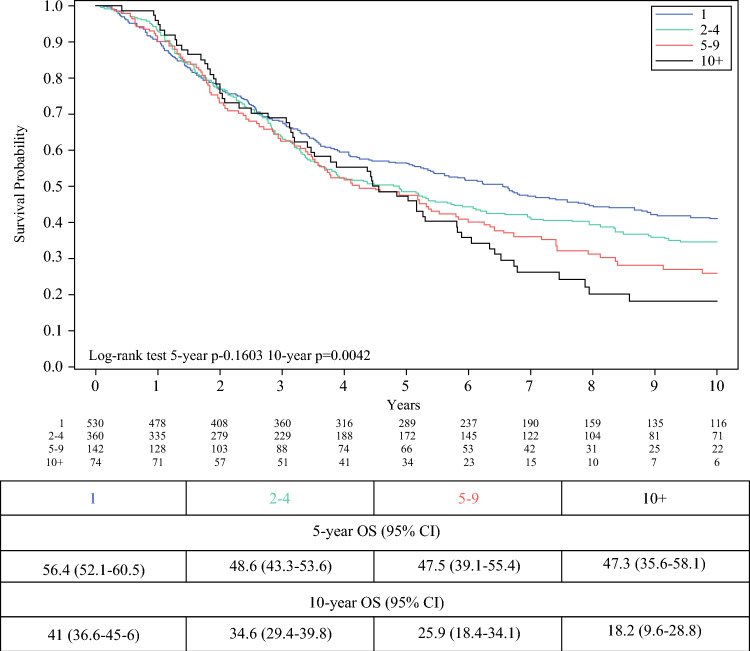
10 year overall survival according to the total number of resected metastases

To ensure that the prognostic analysis accurately reflected the tumor burden, survival also was stratified by the pathologic number of resected metastases (Fig. 3). A post hoc log-rank test on the survival data beyond the 5 year mark demonstrated a statistically significant difference showing a clear separation between the four groups (*p* < 0.05), suggesting that the number of pathologic resected metastases becomes a more prominent prognostic factor as years pass (Fig. 3). In Fig. 2, panel B, we report the 10 year OS curves, starting the survival time by the second resections for the RM group. The adjusted 10 year OS rate for the RM group was 24.9%(95% CI, 17.4–33.1%).

Figure 3 shows the 10 year OS according to the cumulative number of pathologic metastases resected in each patient by all surgical procedures: 41%OS for 1 metastasis, 34.6%OS for 2 to 4 metastases, 25.9%OS for 5 to 9 metastases, and 18.2%OS for 10 or more metastases.

Figure S1 shows 10 year OS stratified by (A) the first resection type and (B) restricted to the MM and RM cohorts. No statistically significant differences were observed between precision (*p* = 0.1700) and other resections (*p* = 0.5662).

Table 2 presents 5 and 10 year OS stratified by histology and the three study cohorts. The patients with germ cell tumor had the highest 10 year OS (72.2%; 95% CI, 60.5–81.0%), followed by kidney (41.8%; 95% CI, 28.1–54.9%), colon-rectum (35.7%; 95%CI, 30.3–41.1%), head and neck (32.4%; 95% CI, 19.8–45.7%), melanoma (31.3%; 95% CI, 22.2–40.9%), and sarcoma (23.9%; 95% CI, 19.0–29.1%). When OS was stratified by the three IRLM classes, a higher 10-year OS was observed for patients with no risk factor (41.9%; 95% CI, 34.6–49.1%) than for patients with one risk factor (38.1%; 95% CI, 33.6–42.6%) or two risk factors (27.9%; 95% CI, 23.1–32.8%) (Fig. S2A). However, when IRLM classes were analyzed in the two major subsets of sarcoma (Fig. S2B) and colorectal cancer (Fig. S2C), the difference in 10 year OS remained statistically significant only for sarcomas (*p* < 0.0001). Similarly, when OS for the three cohorts (SM, MM, RM) was stratified by synchronous and metachronous metastases (Fig. S3A and B), the survival difference lost its significance for patients with synchronous metastases (*p* = 0.0692), possibly due to the small number of patients.

**Table 2:** 5 Year overall survival (OS) stratified by histology

	Total	SM	MM	RM
*5 Year OS (95% CI)*				
Germ cell	75.6 (64.4–83.7)	70 (50.3–83.1)	82.2 (67.4–90.6)	33.3 (0.8–77.4)
Kidney	68.4 (54.7–78.8)	66.7 (46.9–80.5)	58.8 (32.5–77.8)	90 (47.3–98.5)
Colon-rectum	52.9 (47.5–58.0)	60.3 (52.5–67.3)	38.3 (29.7–46.9)	61.8 (47.7–73.1)
Head and neck	52.2 (39.1–64.3)	45.7 (28.9–61.0)	57.1 (33.8–74.9)	—
Sarcoma	41.7 (36.2–47.0)	51.7 (42.4–60.2)	30.4 (22.9–38.2)	47.4 (34.6–59.4)
Melanoma	38.8 (29.2–48.3)	40 (28.9–50.8)	40 (19.3–60.0)	33.3 (0.8–77.4)
*10 Year OS (95% CI)*				
Germ cell	72.2 (60.5–81.0)	70 (50.3–83.1)	76.6 (60.6–86.7)	33.3 (0.8–77.4)
Kidney	41.8 (28.1–54.9)	47.2 (27.8–64.4)	12.2 (0.01–39.9)	60 (25.2–82.7)
Colon-rectum	35.7 (30.3–41.1)	45.1 (37.1–52.9)	26.6 (18.5–35.3)	28.9 (16.8–41.9)
Head and neck	32.4 (19.8–45.7)	30.9 (15.6–47.5)	31.3 (11.6–53.6)	66.6 (5.4–94.5)
Melanoma	31.3 (22.2–40.9)	31.8 (21.3–42.8)	34.3 (14.9–54.8)	—
Sarcoma	23.9 (19.0–29.1)	32.9 (23.9–42.2)	19.4 (12.9–26.8)	19.6 (10.5–30.8)

CI, confidence interval; SM, single metastasis; MM, multiple metastases; RM, recurrent metastases

Finally, a multivariate Cox proportional hazard model, stratified by the three study cohorts, was performed to identify the prognostic factors. In the SM cohort, males had a lower survival than females (hazard ratio [HR], 1.4; 95% CI, 1.1–1.8), patients with a DFI of 36 months or longer had better survival than those with a DFI shorter than 12 months (HR, 0.7; 95% CI, 0.5–0.9), and patients with a primary colon-rectum, germ cell, or other epithelial tumor had better survival than those with sarcoma (*p* = 0.0168, *p* = 0.0047, *p* = 0.022, respectively). Additionally, patients undergoing wedge resections had better survival than those undergoing anatomic procedures (HR, 0.7; 95% CI, 0.5–0.96; Table 3; model A).

**Table 3:** Multivariate Cox proportional hazard model

Risk factors	Model A SM		Model B MM		Model C MR	
	HR (95%CI)	*p* Value	HR (95%CI)	*p* Value	HR (95%CI)	*p* Value
Sex						
Female	1	Ref	1	Ref	1	Ref
Male	**1.4 (1.1–1.8)**	**0.007**	0.9 (0.7–1.1)	0.3072	1 (0.7–1.6)	0.8635
Age (years)						
<50	1	Ref	1	Ref	1	Ref
50–69	1 (0.7–1.4)	0.9166	0.8 (0.6–1)	0.0703	0.9 (0.6–1.5)	0.8084
≥70	1.2 (0.8–1.7)	0.3216	0.8 (0.6–1.3)	0.448	1 (0.5–2)	0.9921
DFI (months)						
0–11	1	Ref	1	Ref	1	Ref
12–35	1 (0.7–1.3)	0.8759	0.9 (0.7–1.2)	0.4696	0.8 (0.5–1.4)	0.4522
36	**0.7 (0.5–0.9)**	**0.0168**	**0.6 (0.4–0.8)**	**0.0005**	0.9 (0.5–1.4)	0.56
Primary tumor site						
Sarcoma	1	Ref	1	Ref	1	Ref
Colon-rectum	**0.7 (0.5–0.9)**	**0.0168**	**0.7 (0.5–1)**	**0.0284**	0.7 (0.4–1.1)	0.1473
Melanoma	1.4 (1–2.1)	0.0655	1 (0.6–1.9)	0.8784	**4.7 (1.2–17.8)**	**0.0225**
Head and neck	1.1 (0.7–1.7)	0.7523	0.8 (0.5–1.5)	0.5778	0.3 (0–1.9)	0.1826
Kidney	0.6 (0.3–1)	0.0719	0.7 (0.4–1.4)	0.3358	**0.4 (0.1–0.9)**	**0.0338**
Uterus	0.6 (0.3–1.3)	0.2028	0.5 (0.3–1.1)	0.0831	0.5 (0.1–2.3)	0.3624
Adenoid cystic carcinoma	2.6 (0.9–7.4)	0.0743	**0.5 (0.3–0.9)**	**0.0266**	0.3 (0.1–1.2)	0.0857
HCC	1.3 (0.7–2.6)	0.4196	1.3 (0.6–2.7)	0.4796	—	0.9762
Other epithelial tumor	**0.5 (0.3–0.9)**	**0.022**	0.7 (0.4–1.4)	0.2848	1 (0.2–4.5)	0.9483
Germ cell	**0.3 (0.2–0.7)**	**0.0047**	**0.1 (0.1–0.2)**	**<.0001**	0.9 (0.2–4.3)	0.9383
First surgical procedure						
Anatomic resection	1	Ref	1	Ref	1	Ref
Wedge	**0.7 (0.5–0.96)**	**0.0234**	0.7 (0.4–1.1)	0.1436	1.2 (0.6–2.3)	0.687
Precision resections	0.9 (0.7–1.2)	0.6044	0.8 (0.6–1.1)	0.2451	1.6 (0.9–2.9)	0.1014
First type of approach						
Thoracotomy	1	Ref	1	Ref	1	Ref
VATS	0.8 (0.6–1)	0.0669	**0.6 (0.4–0.9)**	**0.0118**	1.4 (0.8–2.6)	0.2618
Length of stay at first resection						
No. of pathologic metastases at first resection						
1	–	–	–	–	1	Ref
2–5	–	–	1	ref	**0.6 (0.4–0.9)**	**0.0493**
6+	–	–	1.2 (0.9–1.7)	0.1387	1 (0.5–1.9)	0.9673

Statistically significant results (*p* < 0.05) are indicated in bold type

HR, hazard ratio; CI, confidence interval; DFI, disease-free interval; SM, single metastasis; MM, multiple metastases; RM, repeated metastasectomy

In the MM cohort, patients with a DFI of 36 months or longer had better survival than those with a DFI shorter than 12 months (HR, 0.6; 95% CI, 0.4–0.8). Colon-rectum, adenoid cystic carcinoma, and germ cell tumors had better survival than sarcoma (*p* = 0.0284, *p* = 0.0266, *p* < 0.0001, respectively). Finally, the patients who underwent VATS procedures had better survival than those who underwent thoracotomy procedures (HR, 0.6; 95% CI, 0.4–0.9; Table 3, model B).

In the RM cohort, the patients with melanoma had a worse survival than those with sarcoma (HR, 4.7; 95% CI, 1.2–17.8; Table 3, model C).

### Hospital Stay

The median hospital length of stay (HLS) for the entire cohort was 5 days, with a slight difference across the cohorts (*p* = 0.0032). The patients with SM had a lower median HLS, whereas MM and RM had a higher HLS (Table 1).

More specifically, Table S2 presents the number of patients, the mean time with minimum and maximum HLS values, and the *p* values for comparisons among subsequent resections. The mean HLS time tended to increase with the number of resections, but statistical significance was reached only between the third and the second resections (*p* = 0.0241).

The patients who underwent thoracotomy had a higher HLS than those who had VATS procedures (median, 5 vs. 3 days) in all the three groups, whereas precision resection showed a higher HLS than wedge resection (median, 5 vs. 4 days) in the MM cohort (Table S3).

The patients who underwent thoracotomy had 4.5 to 6 days of HLS across cohorts and resection types (Table S4). In the RM cohort, the patients who received wedge resection by VATS had a median HLS of 2.5 days, compared with 3 days for precision resection and 4.5 days for anatomic resection.

In the multivariate regression analysis (Table S5, model D), precision resection lost its relevance for HLS compared with wedge resection (relative risk [RR], 1.04; *p* = 0.4918). In contrast, VATS maintained a significant association with HLS (RR, 0.60; *p* < 0.0001) as well as the number of metastases (RR, 1.25; *p* = 0.0210) when adjusted for age, sex, resection, approach, and number of metastases.

## Discussion

This study demonstrated that systematic resection of pulmonary metastases by multiple and repeated resections achieved a 35.5%probability of 10 year survival, with a median survival of 5.1 years and a 30-day postoperative mortality of 0.1%in an unselected series of 1106 patients and 1503 consecutive surgical procedures. Even in the less favorable cohort with multiple metastases at the onset (MM), in which the larger tumor burden often correlated with more aggressive disease and increased likelihood of undetectable microscopic lesions, the median survival was 3.8 years and the 10 year OS rate was 30.8%.

On the other hand, the cohort of repeated resections for pulmonary relapse (RM), representing a group of patients with well-controlled primary disease, generally good performance status, and ability to tolerate multiple surgical procedures experienced a median survival of 5.9 years and a 10 year OS of 31.1%. Even after adjustment for time bias, the 10 year OS from the second resection was 24.9%. These results confirm the long-term survival benefit and “potential permanent cure” achieved through multiple and repeated resections in highly selected patients, as reported by other recent studies.^18–20^

The choice of surgical approach and the optimal balance between safe surgical margins and preserved pulmonary function depends on the number, dimension, and depth of all secondary lesions, particularly in recurrent disease. For these reasons, we have always adopted a personalized strategy, ranging from precision resection and wedge excision to segmentectomy, lobectomy, and pneumonectomy, using the most appropriate surgical approach. Although VATS was the first choice for a single lesion or a few peripheral lesions, minimal muscle-sparing lateral thoracotomy was preferred for patients with multiple or central lesions to allow manual palpation of the entire lung and lung-sparing precision resections whenever technically feasible.

In the last 20 years, we have conducted prospective clinical trials of new surgical techniques to improve the effectiveness of precision resection for large and deeply located metastases, including thulium laser and autologous fat-grafting, but the increase in cost and the length of such complex procedures was not justified by the clinical outcome.^21–23^ The finding that anatomic procedures and thoracotomy-based access were associated with lower survival rates in the multivariate analysis should be interpreted with caution. Specifically, anatomic resections (segmentectomy, lobectomy, or pneumonectomy) and thoracotomy were primarily reserved for patients presenting with central, deeper, or larger lesions, as well as those with recurrent intra-lobar relapse. These complex tumor characteristics represent a confounding factor associated with a more aggressive biologic disease and higher metastatic burden, making them the true drivers of the worse prognosis irrespective of the surgical approach. The postoperative course and long-term survival observed in our Institute, as well as in other reported series, prove that precision resection with electrocautery by VATS or minimal open procedure remains a safe and effective option for performing multiple and repeated lung resections with curative intent and adequate local control of disease.^24–29^

Nonetheless, anatomic resections such as segmentectomy, lobectomy, and pneumonectomy represented a non-negligible proportion (28%) of our procedures, which were rewarded by the long-term survival of these carefully selected patients.

In the entire cohort, we observed 5 and 10 year OS rates of 52.1 and 35.5%, respectively, representing a significant improvement in long-term survival compared with that reported by IRLM in 1997 (36 and 26% respectively).^17^ This underscores the substantial progress achieved in systemic therapies for metastatic cancers.

In line with other studies, germ cell tumors showed the highest 5 year OS rate (75.6%),^30^ followed by kidney cancer (68.4%),^31^ with melanoma in the last position (38.8%).^32^ Colorectal cancer remains the most common epithelial tumor for which PM is strongly indicated, and the 5 year OS of 52.9% observed in our study confirms other favorable reports showing 5 year OS of 54 –68%.^33–35^ Sarcomas are the other primary tumors for which current guidelines indicate PM as the primary treatment option for lung metastases due to the low impact of systemic therapy on long-term survival.^36–39^ In our series they accounted for 28.8% of procedures, with a high proportion (43.3%) of multiple metastases and a 5 year OS of 41.7%.

Thanks to the new systemic treatment options, metastatic melanoma represents a rare indication for PM, essentially restricted to solitary lesions.^40^ In our experience, very few patients were treated for multiple metastases, and only three patients were offered repeated surgery.

Stereotactic ablative radiotherapy (SABR) currently is frequently considered for pulmonary oligometastatic disease. According to the European Society for Medical Oncology (ESMO) guidelines, surgery is recommended for resectable colorectal lung metastases, whereas SABR is suggested when surgery is deemed too invasive or the patient is unfit for surgery.^41^ The additional value of SABR versus PM remains uncertain because only a few retrospective studies have compared the two treatments.^42,43^ Lee et al.^43^ analyzed 21 patients receiving SBRT compared with 30 patients undergoing PM, and demonstrated that progression-free survival (PFS) was significantly longer for the patients who underwent PM than for those receiving SBRT. Based on our excellent results, we deem that first-line and repeated PM remain the treatment of choice for fit patients with localized disease.

In the rapidly evolving oncologic landscape, we validated the IRLM prognostic grouping system reported in 1997 with more recent data, reinforcing its relevance in contemporary practice. Our analysis confirmed that long-term survival at 5 and 10 years across the three prognostic groups was statistically different in all histologies (and partially for colon rectal cancer), with significantly worse OS observed for patients presenting with two primary risk factors: (DFI <36 months and multiple metastases).

The current validation underscores the IRLM system’s robustness, but as advancements in treatment options and patient stratification methods emerge, several additional prognostic scoring systems can be tested for pulmonary metastases. They include molecular biomarkers as well as genetic and refined radiomic features, with the aim of capturing a more granular risk profile and guiding more personalized therapeutic approaches.^44–46^

Looking forward, future clinical trials could explore how incorporating such multidimensional scores could further improve the outcome prediction and treatment allocation. Trials might focus on comparing the IRLM with newer models, evaluating outcomes such as PFS and OS in different patient subgroups, and potentially assessing the benefit of new scores integrated into adaptive treatment protocols. Such research could help align prognostic tools with modern oncology practices, ultimately aiming to improve patient outcomes for patients with pulmonary metastases.

This study had some limitations. Its retrospective nature, the single-center experience, and the selection bias within the surgical cohort limit generalizability because we could not compare the outcomes for patients who underwent PM and those for the patients who did not undergo surgery. Additionally, OS was calculated from the date of the first metastasectomy rather than from the date of diagnosis, potentially introducing lead-time bias. Another significant limitation was the lack of detailed information regarding the systemic treatments received by each patient. Furthermore, the low number of patients with lymph node involvement (only 8 [10.1%] of 79 patients who underwent major anatomic resections showed N1 positivity and 11 [3.2%] of 344 of total colorectal patients) did not allow for meaningful further analysis of the prognostic impact of nodal disease. Despite these limitations, the study included a large number of patients and featured long-term follow-up evaluation, making the findings robust.

## Conclusion

In summary, our study demonstrated that repeated PM represents a viable and effective treatment option for selected patients with metastatic disease. The long-term OS rates at 10 years underscore the prospect of “ potential permanent cure” in highly selected cases. Precision pulmonary resections are the optimal technique for multiple metastases to combine parenchymal preservation with adequate local control and enable further resections if new lesions arise. Moreover, an accurate preoperative staging to exclude patients with extrapulmonary disease remains fundamental. As advancements in surgical techniques and systemic therapies continue, the role of PM in combination with other treatments should be further explored. Future studies will be essential to refine patient selection criteria and establish the most effective therapeutic approaches to manage pulmonary metastases in each primary cancer type.

## Supplementary Information

Below is the link to the electronic supplementary material.

Supplementary file1 (DOCX 676 KB)

## Data Availability

Data sharing not applicable to this article as no datasets were generatedor analyzed during the current study.
