# Supplementary material for: Long-Term Results of Multiple Pulmonary Metastasectomies
Source: Ann Surg Oncol. 2026 Feb 11;33(5):4313–23. doi: 10.1245/s10434-025-18992-1 (PMC13083523; doi:10.1245/s10434-025-18992-1)
Supplement: Supplementary file 1 — Supplementary file1 (DOCX 676 KB) [file 10434_2025_18992_MOESM1_ESM.docx]

**Supplementary material**

**Table S1:** Patient’s characteristics

|  | Total N=1106 | Single metastasis (SM) N=530 | Multiple Metastasis (MM) N=426 | Recurrent Metastases (RM) N=150 | p-value |
| --- | --- | --- | --- | --- | --- |
| DEMOGRAPHIC CHARACTERISTICS |  |  |  |  |  |
| Sex - n(%) |  |  |  |  |  |
| Female | 458 (41.4) | 212 (46.3) | 184 (40.2) | 62 (13.5) | 0.6088 |
| Male | 648 (58.6) | 318 (49.0) | 242 (37.3) | 88 (13.6) |  |
| Age - n(%) |  |  |  |  |  |
| <50 | 339 (30.7) | 123 (36.3) | 160 (47.2) | 56 (16.5) | <.0001 |
| 50-69 | 554 (50.1) | 270 (48.7) | 211 (38.1) | 73 (13.2) |  |
| ≥70 | 213 (19.3) | 137 (64.3) | 55 (25.8) | 21 (9.9) |  |
| PRIMARY TUMOR CHARACTERISTICS |  |  |  |  |  |
| Primary tumor site - n(%) |  |  |  |  |  |
| Sarcoma | 319 (28.8) | 120 (37.6) | 138 (43.3) | 61 (19.1) | <.0001 |
| Colon-rectum | 344 (31.1) | 169 (49.1) | 120 (34.9) | 55 (16.0) |  |
| Melanoma | 98 (8.9) | 75 (76.5) | 20 (20.4) | 3 (3.1) |  |
| Head and neck | 59 (5.3) | 35 (59.3) | 21 (35.6) | 3 (5.1) |  |
| Kidney | 58 (5.2) | 30 (51.7) | 18 (31) | 10 (17.2) |  |
| Uterus | 38 (3.4) | 20 (52.6) | 16 (42.1) | 2 (5.3) |  |
| Adenoid cystic carcinoma | 31 (2.8) | 6 (19.4) | 19 (61.3) | 6 (19.4) |  |
| HCC | 29 (2.6) | 14 (48.3) | 11 (37.9) | 4 (13.8) |  |
| Other epithelial^1^ | 52 (4.7) | 31 (59.6) | 18 (34.6) | 3 (5.8) |  |
| Germ Cell | 78 (7.1) | 30 (38.5) | 45 (57.7) | 3 (3.8) |  |
| PRIMARY PULMONARY RESECTION CHARACTERISTICS |  |  |  |  |  |
| DFI - n(%) |  |  |  |  |  |
| 0-11 | 392 (35.4) | 163 (41.6) | 178 (45.4) | 51 (13.0) | 0.0014 |
| 12-35 | 350 (31.6) | 165 (47.1) | 133 (38) | 52 (14.9) |  |
| 36 | 364 (32.9) | 202 (55.5) | 115 (31.6) | 47 (12.9) |  |
| Firs surgical approach - n(%) |  |  |  |  |  |
| Unilateral | 899 (81.3) | 530 (59.0) | 249 (27.7) | 120 (13.3) | <.0001 |
| Bilateral | 207 (18.7) | 0 | 177 (85.5) | 30 (14.5) |  |
| First Type of approach - n(%) |  |  |  |  |  |
| Thoracotomy | 852 (77.0) | 363 (42.6) | 367 (43.1) | 122 (14.3) | <.0001 |
| VATS | 240 (21.7) | 164 (683) | 51 (21.3) | 25 (10.4) |  |
| Other^2^ | 14 (1.3) | 3 (21.4) | 8 (57.1) | 3 (21.4) |  |
| First surgical procedure - n(%) |  |  |  |  |  |
| Precision Resections | 547 (49.5) | 171 (31.3) | 295 (53.9) | 81 (14.8) | <.0001 |
| Wedge | 243 (22.0) | 165 (67.9) | 41 (16.9) | 37 (15.2) |  |
| Lobectomy | 165 (14.9) | 109 (65.7) | 40 (24.2) | 16 (9.6) |  |
| Segmentectomy | 139 (12.6) | 76 (54.7) | 47 (33.8) | 16 (11.5) |  |
| Pneumonectomy | 12 (1.1) | 9 (75.0) | 3 (25.0) | 0 |  |
| Length of stay - n(%) |  |  |  |  |  |
| <7 days | 868 (78.5) | 421 (48.5) | 329 (37.9) | 118 (13.6) | 0.7108 |
| ≥7 days | 238 (21.5) | 109 (45.8) | 97 (40.8) | 32 (13.4) |  |
| median (IQR) | 5 (4-6) | 5 (3-6) | 5 (4-6) | 5 (4-6) |  |
| OTHER CHARACTERISTICS |  |  |  |  |  |
| IRLM^3^ risk factors: |  |  |  |  |  |
| No risk factors | 223 (20.2) | 202 (90.6) | 0 | 21 (9.4) | <.0001 |
| One risk factor | 513 (46.4) | 328 (63.9) | 116 (22.6) | 69 (13.5) |  |
| Two risk factors | 370 (33.5) | 0 | 310 (83.8) | 60 (16.2) |  |

^1^Other epithelial: Mediastinum, Ovary, Pancreas, Prostate, Stomach, Adrenal, Penis, Bladder, Esophagus, Mandibular adamantinoma, Anal canal carcinoma, Thyroid

^2^Other= Clamshell; Transmanubrial; Sternotomy

^3^ International Registry of Lung Metastases; No risk factors (DFI ≥ 36 months, and single metastasis); One risk factor (DFI < 36 months or multiple metastases); Two risk factors (DFI < 36 months and multiple metastases)

**Table S2:** Median and mean values of hospital stay stratified by resections

|  | **N** | **Median** | **IQR** | **Mean** | **p-value** | |
| --- | --- | --- | --- | --- | --- | --- |
| First resection | 1300 | 5 | 4-6 | 5.6 |  |  |
| Second resection | 165 | 5 | 4-7 | 5.9 | second vs. first | 0.0872 |
| Third resection | 33 | 6 | 5-10 | 9.7 | third vs. second | 0.0241 |
| Fourth resection | 4 | 6.5 | 4.5-23.5 | 14 | fourth vs. third | 0.8633 |
| Fifth resection | 1 | 10 |  | 10 | fifth vs. fourth | 0.4795 |

**Table S3:** Hospital stays according to the three study cohorts and surgical approach and types

|  |  | Total N=1503 | Hospital stay -  median (IQR) | >= 7 days | P.value |
| --- | --- | --- | --- | --- | --- |
| SM | Wedge | 165 | 4 (3-5) | 13 (7.8) | 0.0322^a^ |
|  | Precision resection | 171 | 4 (3-6) | 31 (18.1) | 0.0054^b^ |
|  | Anatomical resection | 194 | 6 (5-7) | 65 (33.5) |  |
|  | Thoracotomy | 363 | 5 (4-7) | 96 (26.5) | <.0001^c^ |
|  | VATS | 164 | 3 (2-4) | 11 (6.7) | <.0001^d^ |
| MM | Wedge | 46 | 4 (3-5) | 3 (6.5) | 0.0004^a^ |
|  | Precision resection | 423 | 5 (4-6) | 86 (20.3) | 0.0233^b^ |
|  | Anatomical resection | 126 | 6 (5-7) | 47 (37.3) |  |
|  | Thoracotomy | 519 | 5 (4-6) | 126 (24.3) | <.0001^c^ |
|  | VATS | 68 | 3 (2-4) | 5 (7.4) | 0.0016^d^ |
| RM | Wedge | 73 | 5 (3-6) | 18 (24.7) | 0.6002^a^ |
|  | Precision resection | 205 | 5 (4-6) | 37 (18.1) | 0.2235^b^ |
|  | Anatomical resection | 100 | 6 (5-8) | 41 (41.0) |  |
|  | Thoracotomy | 334 | 5 (4-7) | 90 (26.9) | <.0001^c^ |
|  | VATS | 40 | 3 (2-4) | 3 (7.5) | 0.0065^d^ |

^a^ Median hospital stay between precision and wedge resections

^b^ Percentage of Hospital stays between precision and wedge resections

^c^ Median hospital stay between thoracotomy and VATS

^d^ Percentage of Hospital stays between thoracotomy and VATS

**Table S4:** Hospital median stratified by the surgical approach and type in the three study cohorts.

|  |  | Wedge  n (median) | Precision resection  n (median) | Anatomical resection n (median) |
| --- | --- | --- | --- | --- |
| SM | Thoracotomy | 96 [5] | 110 [5] | 157 [6] |
|  | VATS | 69 [3] | 59 [3] | 36 [4] |
| MM | Thoracotomy | 36 [4.5] | 375 [5] | 108 [6] |
|  | VATS | 10 [3] | 43 [3] | 15 [3] |
| RM | Thoracotomy | 60 [5] | 181 [5] | 93 [6] |
|  | VATS | 11 [2.5] | 22 [3] | 6 [4.5] |

**Table S5:** Generalized linear models for hospital length of stay

|  | Model A | Model B | Model C | Model D |
| --- | --- | --- | --- | --- |
|  | RR (95% CI) | RR (95% CI) | RR (95% CI) | RR (95% CI) |
|  | P-Value | P-Value | P-Value | P-Value |
|  |  |  |  |  |
| **Resection** |  |  |  |  |
| Wedge | ref |  |  | ref |
| Precision resection | 1.16 (1.05-1.29) **0.0039** |  |  | 1.04 (0.93-1.17) 0.4918 |
|  |  |  |  |  |
| **Approach** |  |  |  |  |
| Thoracotomy |  | ref |  | ref |
| VATS |  | 0.57 (0.53-0.62) **<.0001** |  | 0.60 (0.55-0.65) **<.0001** |
|  |  |  |  |  |
| **Number of metastases** |  |  |  |  |
| 1 |  |  | ref | ref |
| 2-5 |  |  | 1.14 (1.04-1.26) **0.0049** | 1.03 (0.93-1.15) 0.5818 |
| 6+ |  |  | 1.44 (1.21-1.73) **<.0001** | 1.25 (1.03-1.51) **0.0210** |

Model A: Adjusted for Sex, Age and Resection

Model B: Adjusted for Sex, Age, and Approach

Model C: Adjusted for Sex, Age, and Number of metastases

Model D: Adjusted for Sex, Age, Resection, Approach, Number of metastases

**
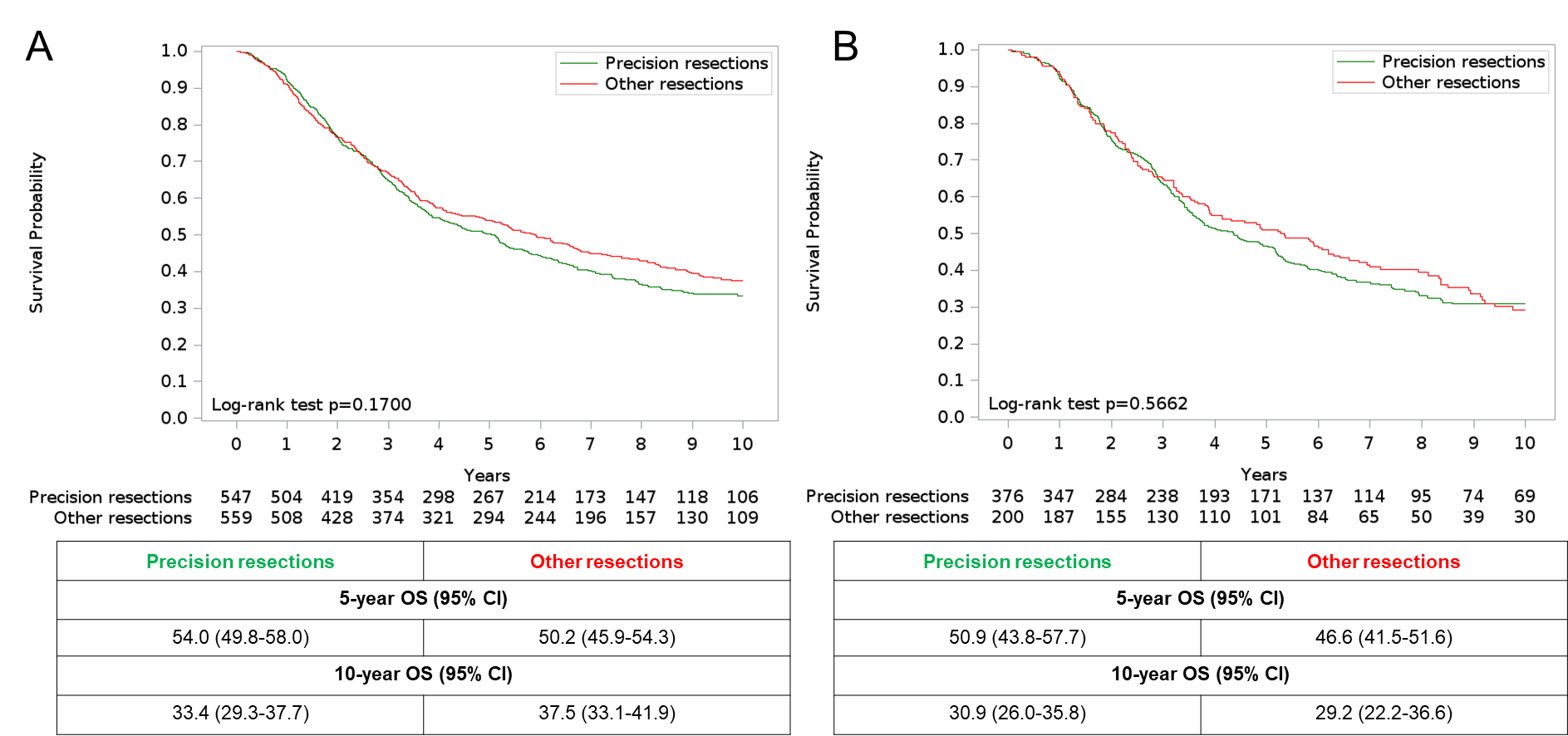
Figure S1:** 10-years OS according to the resection type (A); stratified only in the MM and RM cohorts (B)

**Figure S2.** 10-years OS according to the IRLM risk group (A); stratified by Sarcoma (B) and Colon-rectum (C)


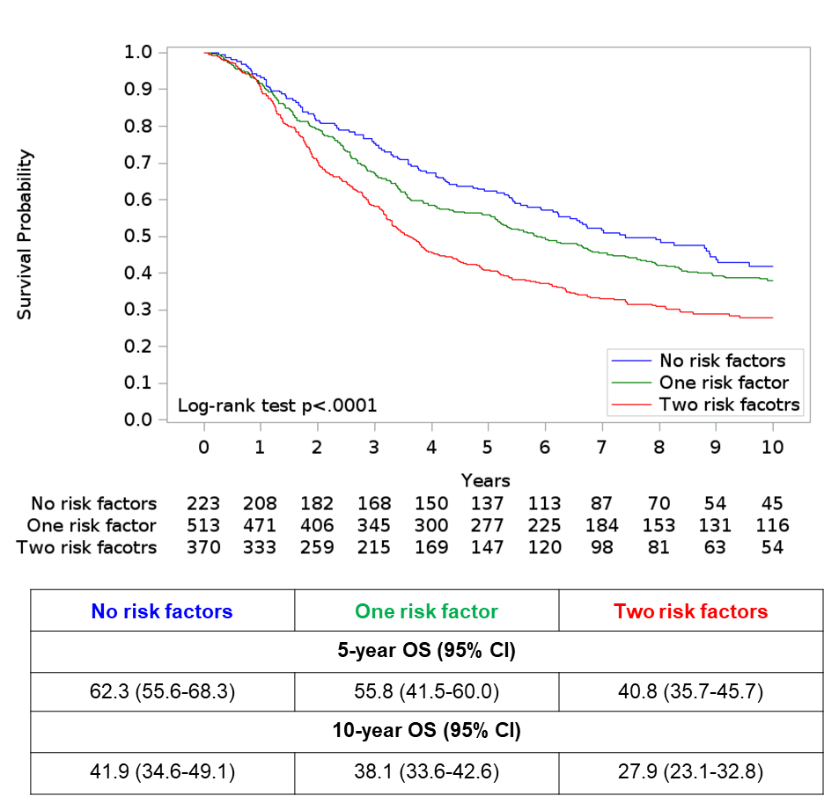


C

A

A


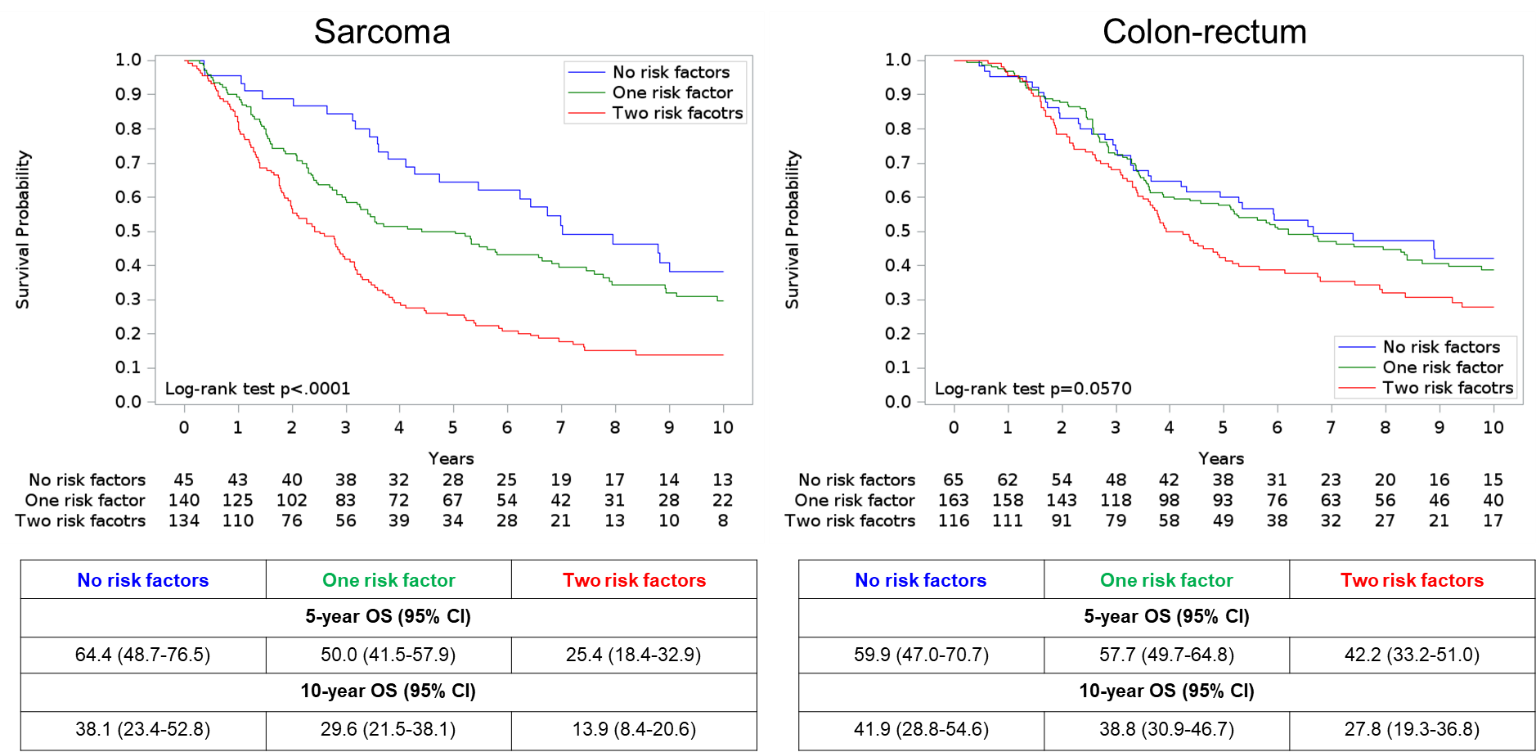


B

**Figure S3.** 10-year overall survival according to the three cohorts stratified by Synchronous (A) and Metachronous (B)

**
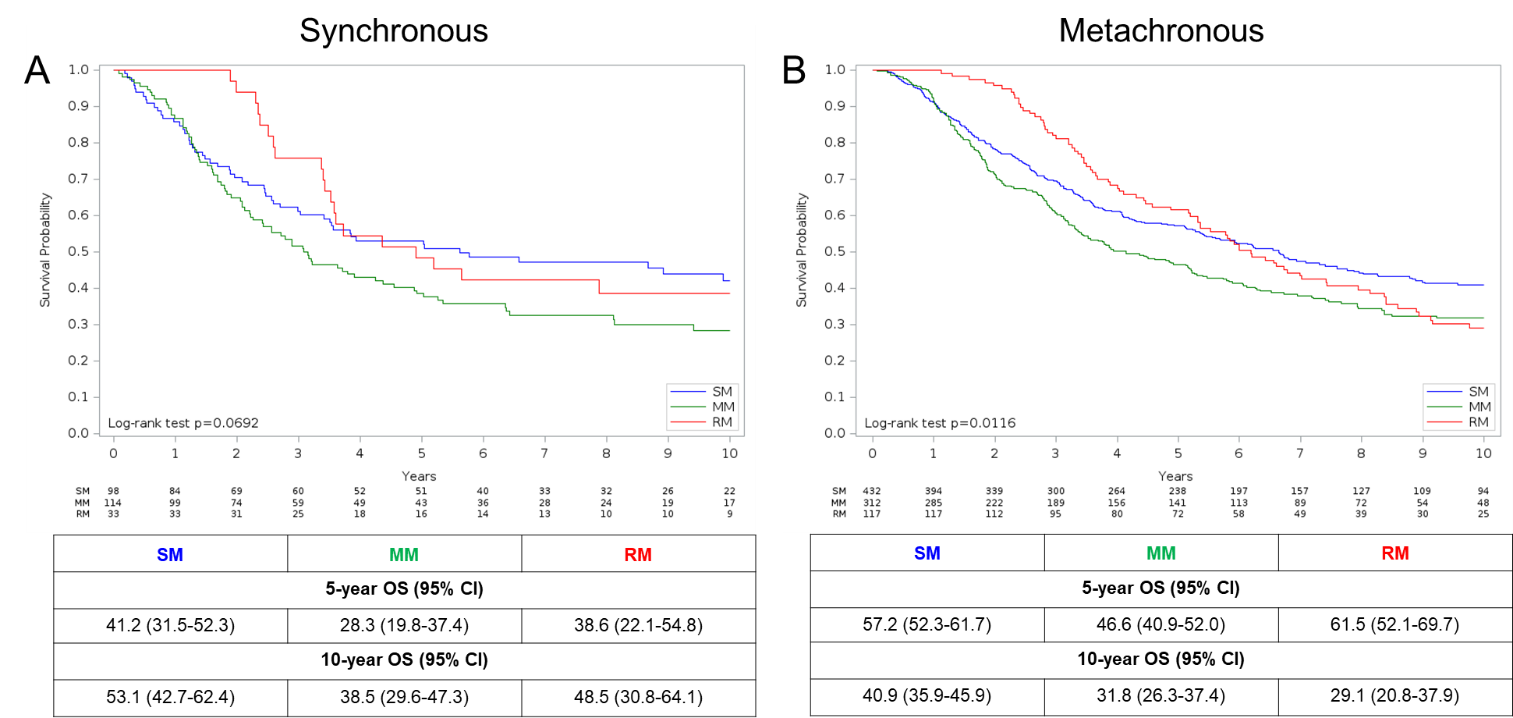
**
